# Cyclosporine Broadens the Therapeutic Potential of Lenalidomide in Myeloid Malignancies

**DOI:** 10.33696/immunology.2.049

**Published:** 2020

**Authors:** Aixia Dou, Jing Fang

**Affiliations:** 1 Department of Drug Discovery and Biomedical Sciences, University of South Carolina College of Pharmacy, Columbia, SC, USA

## Abstract

The immunomodulatory drug lenalidomide is used for the treatment of certain hematologic malignancies, including myelodysplastic syndromes (MDS). Lenalidomide interacts with cereblon (CRBN), a component of the CRL4^CRBN^ E3 ubiquitin ligase complex, leading to ubiquitination and subsequent degradation of substrates, such as transcription factor Ikaros (Ikaros family zinc finger 1, IKZF1). With a genome loss of function screen, we recently identified two novel pathways mediated by lenalidomide in MDS. In this review, we summarized the major findings of these two pathways and their clinical implications. Depletion of G protein-coupled receptor 68 (GPR68) or an endogenous calcineurin (CaN) inhibitor, regulator of calcineurin 1 (RCAN1), reversed the inhibitory effect of lenalidomide on MDSL cells, an MDS cell line. Intriguingly, both GPR68 and RCAN1 expression levels were upregulated in MDSL cells after treatment with lenalidomide that was dependent on diminishment of IKZF1, indicating that IKZF1 functioned as a transcription repressor for GPR68 and RCAN1. Mechanistic studies revealed that upregulation or activation of GPR68 induced a Ca^2+^/calpain pro-apoptotic pathway, while upregulation of RCAN1 inhibited the CaN pro-survival pathway in MDSL cells. Notably, the pharmacological CaN inhibitor, cyclosporine, enhanced the sensitivity to lenalidomide in MDS as well as acute myeloid leukemia (AML). Surprisingly, pretreatment with lenalidomide reversed the immunosuppressive effects of cyclosporine on T lymphocytes. Our studies suggest that lenalidomide mediates degradation of IKZF1, leading to derepression of GPR68 and RCAN1 that activates the Ca2+/calpain pro- apoptotic pathway and inhibits the CaN pro-survival pathway, respectively. Our studies implicate that cyclosporine extends the therapeutic potential of lenalidomide to myeloid malignancies without compromising immune function.

## Introduction

Thalidomide, lenalidomide and pomalidomide are synthetic immunomodulatory drugs (IMiDs) that have recently drawn attention in both clinics and basic research [1]. Thalidomide was synthesized from glutamic acid and was banned due to its teratogenicity in pregnant women [1]. Lenalidomide is a 4-amino-glutamyl analogue of thalidomide and is approved for the treatment of certain hematologic malignancies. Lenalidomide is used for the treatment of lower-risk red blood cell (RBC) transfusion-dependent myelodysplastic syndromes (MDS) with deletion of chromosome 5q (del(5q)) with or without additional cytogenetic abnormalities [2–4]. MDS patients with del(5q) exhibit much higher hematologic and cytogenetic responses than those without del(5q) [3–6]. In contrast to lower-risk MDS patients, the response to lenalidomide monotherapy is poor in patients with higher-risk del(5q) MDS and acute myeloid leukemia (AML), especially in those with TP53 mutations [7,8]. Therefore, lenalidomide in combination with other drugs are being evaluated. Indeed, better responses are observed in patients with higher-risk del(5q) MDS and AML who are treated with lenalidomide in combination with hypomethylating agent azacitidine than lenalidomide monotherapy [8–11]. Despite a high response to lenalidomide in lower-risk del(5q) MDS, half of the patients relapse within 2–3 years, which may be associated with the malignant MDS stem cells [5,12,13]. Accumulating evidence implicate that lenalidomide selectively inhibits the del(5q) clone, which is associated with modulation of several haploinsufficient genes that are localized on the deleted 5q regions, such as cell division cycles 25C (CDC25C) and protein phosphatase 2 phosphatase activator (PTPA, also known as PP2A), secreted protein acidic and cysteine rich (SPARC), ribosomal protein S14 (RPS14) and miR-145 [14–21].

In addition to the direct effects on the pathological MDS clones, lenalidomide also exerts pleiotropic effects on immune cells [22,23]. In response to lipopolysaccharide (LPS), peripheral blood mononuclear cells (PBMC) secreted less tumor necrosis factor alpha (TNFα), interleukin 1 beta (IL-1β) and interleukin 6 (IL-6) but more interleukin 10 (IL-10) in the presence of lenalidomide [24]. T cells express T cell receptor (TCR) and coreceptors (i.e. CD4 and CD8) that recognize the antigen peptides presented by major histocompatibility complex (MHC) on antigen presenting cells (APC). Upon recognizing antigen/MHC complex, TCR and coreceptors, together with the co- stimulatory signal provided by CD28 on T cells and B7 on APC, activate signaling pathways that lead to proliferation, survival, differentiation, cytokine secretion, expression of cytokine receptors and cytotoxicity of T cells [25]. Lenalidomide is shown to directly activate CD28 and the subsequent signaling pathways, resulting in secretion of interleukin 2 (IL-2) and interferon gamma (IFNγ) [26,27]. In addition, lenalidomide, in combination with dexamethasone, is approved for the treatment of multiple myeloma (MM) that is associated with its immunomodulatory effects [1,24,26–33].

Recently, cereblon (CRBN) is identified as a primary target that directly binds IMiDs and mediates the teratogenic and anti-tumor activities [34–36]. CRBN, together with CUL4, DDB1 and ROC1, forms the CRL4^CRBN^ E3 ubiquitin ligase complex with CRBN as the substrate adaptor [34,37,38]. In the presence of lenalidomide, CRBN binds several proteins, such as Ikaros (Ikaros family zinc finger 1, IKZF1), Aiolos (Ikaros family zinc finger 3, IKZF3) and casein kinase 1 isoform alpha (CSNK1A1, also known as CK1α), leading to ubiquitination and subsequent degradation of these substrates by proteasome[39–42]. In order to better understand the mechanism of action of lenalidomide in MDS cells, we performed a genome-wide RNA interference screen and identified novel signaling pathways that modulated the sensitivity to lenalidomide in MDS/AML [43,44]. Here we summarized the major discoveries about the newly discovered signaling pathways mediated by lenalidomide in MDS/AML. Our studies provide insights into rational combinatorial therapy of lenalidomide in myeloid malignancies.

## Lenalidomide regulates the GPR68/Ca^2+^/calpain pathway in MDS

In order to understand the mechanism of action of lenalidomide in MDS, we characterized the effects of lenalidomide on an MDS cell line, MDSL cells. The MDSL cells, initially derived from a low-risk MDS patients with del(5q) [45,46], contained a mixed populations of CD34^+^ and CD34^−^ cells that behaved differentially in response to lenalidomide. In the presence of lenalidomide, the CD34^−^ MDSL cells exhibited more Annexin V^+^ cells, while the CD34^+^ MDSL cells formed fewer colonies in semi-solid methylcellulose. In liquid culture, both CD34^+^ and CD34^−^ MDSL cells grew less efficiently in the presence of lenalidomide. Our findings suggested that lenalidomide exerted pleiotropic inhibitory effects on MDSL cells, including inhibition on growth, survival and clonogenicity. Intriguingly, depletion of CRBN reversed the inhibitory effects of lenalidomide on MDSL cells, indicating that lenalidomide acted through the CRL4^CRBN^ E3 ubiquitin ligase complex.

In order to identify the genes that were critical for lenalidomide-mediated inhibitory effects on MDSL cells, we performed a genome-wide RNA interference screen in MDSL cells [43]. We found that depletion of a G protein-coupled receptor, GPR68, reversed the inhibitory effects of lenalidomide on MDSL cells. In addition, GPR68 mRNA and protein levels were upregulated in MDSL cells after treatment with lenalidomide. Among the identified targets of the CRL4^CRBN^ E3 ubiquitin ligase complex (i.e. IKZF1, IKZF3 and CK1α), the promoter region of GPR68 gene locus contained binding peaks for IKZF1, indicating that IKZF1 may regulate GPR68 expression. As expected, depletion of IKZF1 increased GPR68 expression, while overexpression of wild type IKZF1, but not degradation-resistant IKZF1, reduced GPR68 expression in MDSL cells, indicating that IKZF1 acted as a transcription repressor, repressing GPR68 expression. These results suggested that in the presence of lenalidomide, IKZF1 was degraded by the CRL4^CRBN^ E3 ubiquitin ligase/proteasome system, leading to derepression of GPR68 in MDSL cells.

In response to extracellular protons or overexpression, GPR68 undergoes conformational changes, leading to association with G proteins (Gq/11) and subsequent inositol phosphate formation and cytosolic calcium (Ca^2+^) accumulation [47]. Further studies revealed that upregulation of GPR68 in MDSL cells upon treatment with lenalidomide led to accumulation of cytosolic Ca^2+^ ions that was required for lenalidomide-mediated inhibitory effect on MDSL cells. Screening of pharmacological inhibitors that targeted Ca^2+^ -related signaling pathways revealed that inhibition of calpain reversed apoptosis in MDSL cells after treatment with lenalidomide, indicating that lenalidomide activated a Ca^2+^/calpain pro-apoptotic pathway in MDSL cells through derepressing GPR68. Notably, 3,5-disubstituted isoxazoles (Isx), a GPR68 agonist [48], significantly increased cytosolic Ca^2+^ levels and apoptosis and reduced colony formation in MDSL cells in the presence of lenalidomide, indicating that both overexpression and activation of GPR68 could enhance lenalidomide-mediated inhibitory effects on MDSL cells.

## Lenalidomide regulates the RCAN_1_/CaN pathway in MDS

Despite our observation in that GPR68 agonist Isx enhanced the inhibitory effects of lenalidomide on MDSL cells through inducing a Ca^2+^/calpain pro-apoptotic pathway, Isx has not been approved by the U.S. Food and Drug Administration (FDA) for any clinical applications. This prompted us to look for alternative candidates that could enhance the sensitivity to lenalidomide in MDS. From the genome-wide RNA interference screen in MDSL cells, we found that depletion of regulator of calcineurin 1 (RCAN1) also reversed the inhibitory effect of lenalidomide on MDSL cells. RCAN1 is the endogenous inhibitor of the serine/threonine phosphatase calcineurin (CaN) [49], a critical signaling molecule during T cell activation [50,51]. The pharmacological inhibitor of CaN, cyclosporine, is an FDA-approved drug that is used to prevent immune rejection after organ transplantation [52]. We therefore examined the effect of the RCAN1/CaN pathway on the sensitivity to lenalidomide in MDS.

Similar to GPR68, RCAN1 mRNA and protein levels were also upregulated in MDSL cells after treatment with lenalidomide. Intriguingly, depletion of IKZF1 also increased RCAN1 expression in MDSL cells, indicating that IKZF1 acted as a transcription repressor, repressing RCAN1 expression as well. These data suggested that through degrading IKZF1, lenalidomide derepressed the expression of both GPR68 and RCAN_1_ in MDSL cells. In contrast to GPR68, we failed to find any obvious binding peaks for IKZF1 in the promoter region of RCAN1 gene locus, indicating that IKZF1 may regulate RCAN1 expression through a different mechanism. Indeed, recent studies implicate IKZF1 functions as a tumor suppressor in T cell leukemia via global regulation of the enhancer/super-enhancer landscape [53].

Consistent with the function of RCAN1 in T cells, depletion of RCAN1 in MDSL cells resulted in increased activity of CaN, indicating that RCAN1 also functioned as an inhibitor of CaN in MDSL cells. To understand the function of the RCAN1/CaN pathway in MDSL cells, we used cyclosporine to inhibit CaN activity. As expected, treatment of cyclosporine resulted in increased activity of CaN. Consistent with the function of CaN in T cells [50,51], treatment of cyclosporine resulted in increased Annexin V^+^ cells in MDSL cells, indicating that CaN was constitutively activated and provided a pro-survival signal in MDSL cells. Our results suggested that in addition to inducing the GPR68/Ca^2+^/calpain pro-apoptotic pathway, lenalidomide also inhibited the CaN pro-survival pathway via derepressing RCAN1 expression in MDSL cells. Lenalidomide crosslinked the GPR68/Ca^2+^/calpain and the RCAN1/CaN pathways through degrading IKZF1.

## Cyclosporine enhances the sensitivity to lenalidomide in MDS

Given that CaN provided a pro-survival signal in MDSL cells, we examined the effect of cyclosporine on the sensitivity to lenalidomide in MDS. We pretreated MDSL cells with control or lenalidomide, followed by co-treatment with control or cyclosporine. Co-treatment with lenalidomide and cyclosporine induced more Annexin V^+^ cells than single treatment with lenalidomide or cyclosporine in MDSL cells. In addition, MDSL cells grew far fewer colonies in the presence of lenalidomide and cyclosporine than in the presence of lenalidomide only in methylcellulose. These results suggested that cyclosporine enhanced the sensitivity to lenalidomide in MDSL cells. In addition, we examined the effect of cyclosporine on the sensitivity to lenalidomide in primary bone marrow samples from MDS patients. We found more Annexin V^+^ cells in two MDS specimens after co-treatment with lenalidomide and cyclosporine than single treatment with lenalidomide or cyclosporine. Notably, one of the MDS patients was diagnosed with RAEBII, higher-risk MDS, indicating that cyclosporine enhanced the sensitivity to lenalidomide in both lower- and higher-risk MDS.

## Cyclosporine enhances the sensitivity to lenalidomide in AML

We next examined the effect of cyclosporine on the sensitivity to lenalidomide in AML. TF-1 cells are a del(5q) AML cell line that is sensitive to lenalidomide. Similar to MDSL, co-treatment of lenalidomide and cyclosporine significantly increased Annexin V^+^ cells in TF-1 cells compared to single treatment with lenalidomide or cyclosporine. In addition, we examined the effect of cyclosporine on the sensitivity to lenalidomide in AML patient-derived xenograft (PDX) models. All three PDX models were derived from pediatric AML after relapse who failed to respond to chemotherapy [54]. Among the three PDX models, one was sensitive to lenalidomide as evidenced by increased Annexin V^+^ after treatment with lenalidomide. Co-treatment with lenalidomide and cyclosporine induced more Annexin V^+^ cells in the lenalidomide-sensitive PDX model than single treatment with lenalidomide or cyclosporine. Surprisingly, co-treatment with lenalidomide and cyclosporine induced apoptosis in the two PDX models that were resistant to lenalidomide. Intriguingly, the lenalidomide-sensitive PDX model contained wild type p53, while the lenalidomide-resistant PDX models contained mutant p53, which was consistent with clinical observations in that p53 mutation was associated with resistance to lenalidomide [2,8]. In addition, the three PDX models contained MLL arrangements and complex karyotypes, indicating that cyclosporine enhanced the sensitivity to lenalidomide in AML irrespective of the cytogenetic aberrations.

## Pretreatment of lenalidomide reverses the immunosuppressive effect of cyclosporine

Upon recognizing the antigen/MHC complex, TCR, coreceptors and co-stimulation activate a series of signaling pathways, among which CaN and the subsequent nuclear factor of activated T cells (NFAT) play a critical role during T cell activation. The CaN/NFAT pathway promotes the production of IL-2, resulting in proliferation and survival of T cells [55,56]. After organ transplantation, T cells play a major role mediating immune rejection. In clinics, high-dose cyclosporine is used to prevent immune rejection through inhibiting the CaN/NFAT pathway and T cell response [57,58]. In contrast, low-dose cyclosporine is used in miscellaneous pathological disorders [59–61]. Abnormal immune function is also implicated in the pathogenesis of MDS [62–64]. In lower-risk MDS, autologous T cells mediate apoptosis in both MDS cells and normal hematopoietic cells [65]. In higher-risk MDS, T cells fail to recognize antigen/MHC complex on APC due to inhibitory signals, such as programmed death-1 (PD-1) and its ligand programed death ligand 1 (PD-L1), leading to defective tumor surveillance [66]. Therefore, immunosuppressive therapy, such as cyclosporine, is used for patients with lower-risk MDS, while immune checkpoint inhibitors are used for patients with higher-risk MDS [64,67–69]. We examined the combined effects of lenalidomide and cyclosporine on T cell response. T cells were harvested from spleens of C57Bl6 mice and activated in the presence of anti-CD3 and anti-CD28 antibodies. T cell activation was inhibited by co-treatment with lenalidomide and cyclosporine but not single treatment with lenalidomide, indicating that cyclosporine inhibited T cell activation. Intriguingly, when we pretreated T cells with lenalidomide, co-treatment with lenalidomide and cyclosporine didn’t inhibit T cell activation, indicating that pretreatment of lenalidomide reversed the inhibitory effect of cyclosporine on T cell activation. Lenalidomide directly binds human CRBN but not mouse Crbn due to a mutation within the binding domain [41]. Our results indicated that lenalidomide reversed the immunosuppressive effect of cyclosporine through a CRBN-independent manner. Lenalidomide-mediated direct activation of CD28 may explain the reversion of cyclosporine’s immunosuppressive effect on T cells, which needs further clarification.

## Conclusion

**Figure 1:**
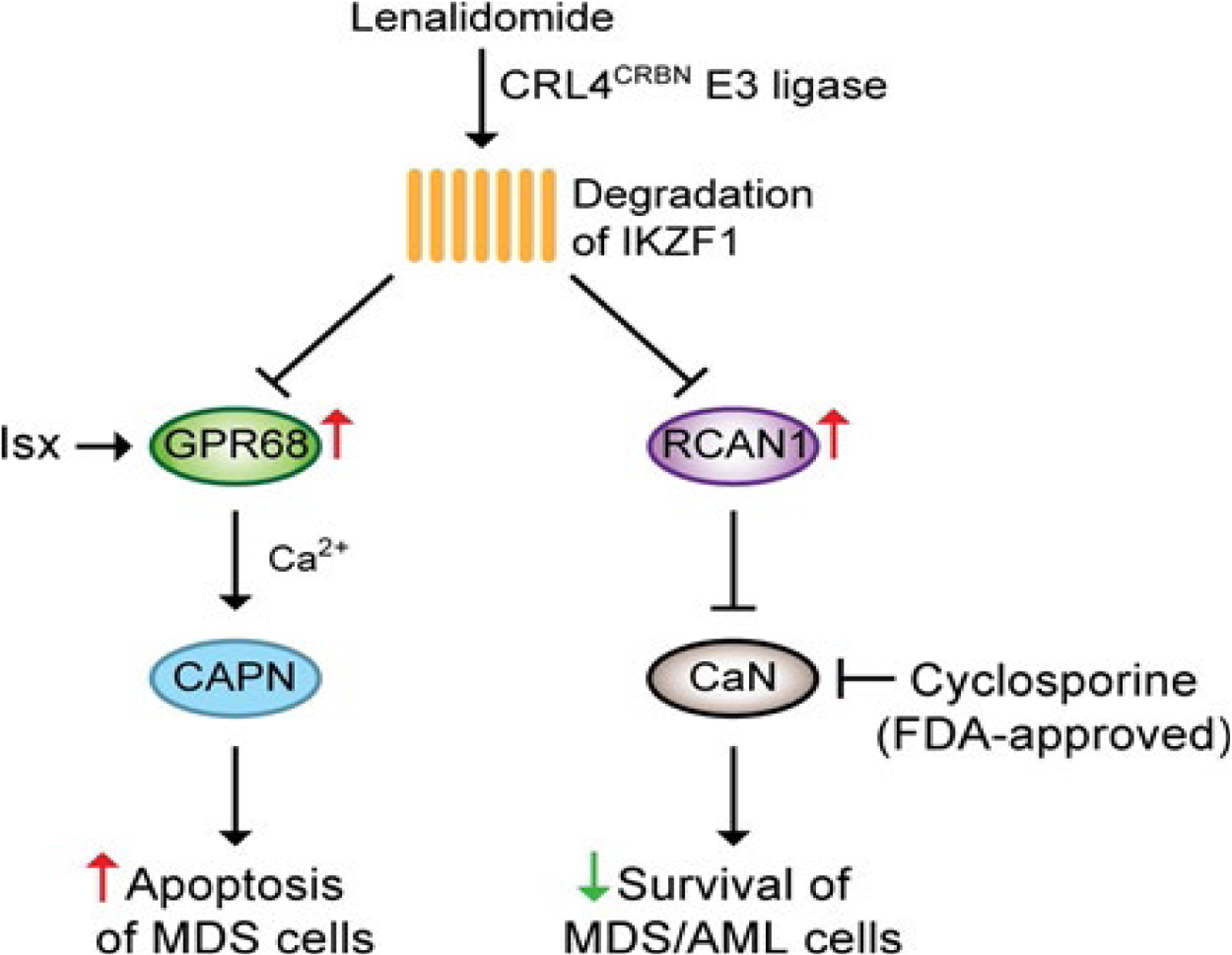
Overview of the mechanism of action of lenalidomide in MDS/AML. Through the CRL4^CRBN^ E3 ligase complex, lenalidomide mediates degradation of IKZF1, leading to derepression of GPR68 and RCAN1. Derepression or activation (i.e. Isx) of GPR68 induces a Ca^2+^/calpain (CAPN) pro-apoptotic pathway. Derepression of RCAN1 inhibited the CaN pro-survival pathway. Cyclosporine enhances the sensitivity to lenalidomide in MDS/AML through inhibiting CaN activity.

Our recent studies identify that lenalidomide mediates degradation of IKZF1, leading to derepression of GPR68 and RCAN1 (Figure 1). Upregulation of GPR68 activates a Ca^2+^/calpain pro-apoptotic pathway in MDS cells. In addition, GPR68 agonist Isx also activates the Ca^2+^/calpain pro-apoptotic pathway, thus enhancing the cytotoxicity of lenalidomide in MDS. However, the fact that Isx is not an FDA-approved drug limits its clinical application. Upregulation of RCAN1 inhibits the CaN pro-survival pathway in MDS cells. The pharmacological inhibitor of CaN, cyclosporine, induces apoptosis in MDS/AML cells, thus enhancing the cytotoxicity of lenalidomide in MDS/AML. Surprisingly, pretreatment of lenalidomide reverses the immunosuppressive effect of cyclosporine on T cells. Our studies provide the rational therapeutic combination of lenalidomide and cyclosporine in myeloid malignancies.
